# Supplementary figures and images for: Small molecule inhibitor of PPARγ acetylation promotes insulin sensitization and browning of white adipose tissue with improved safety
Source: bioRxiv. 2025 Dec 16:2025.12.15.694265. Preprint. [Version 1] doi: 10.64898/2025.12.15.694265 (PMC12746064; doi:10.64898/2025.12.15.694265)

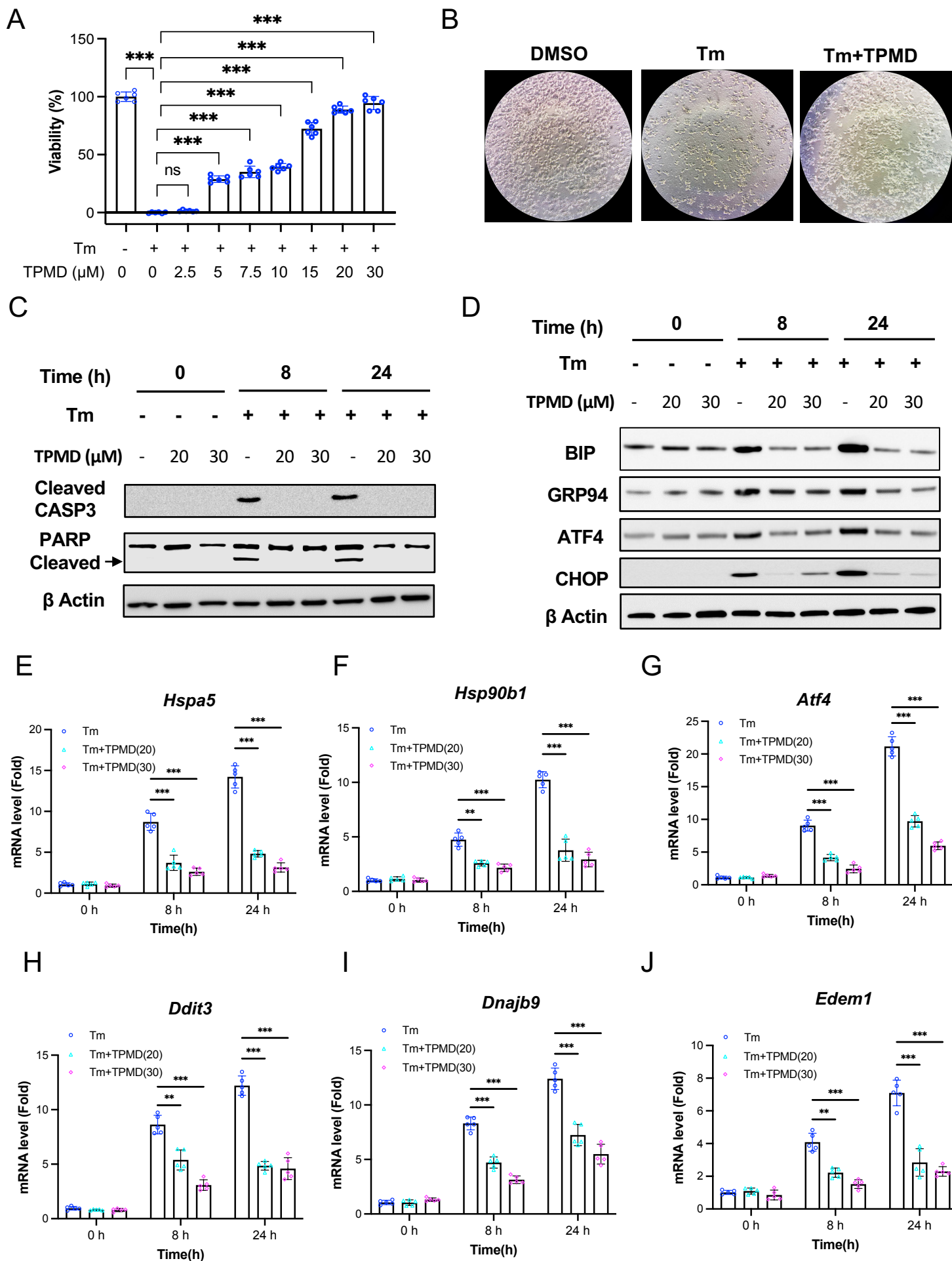

A

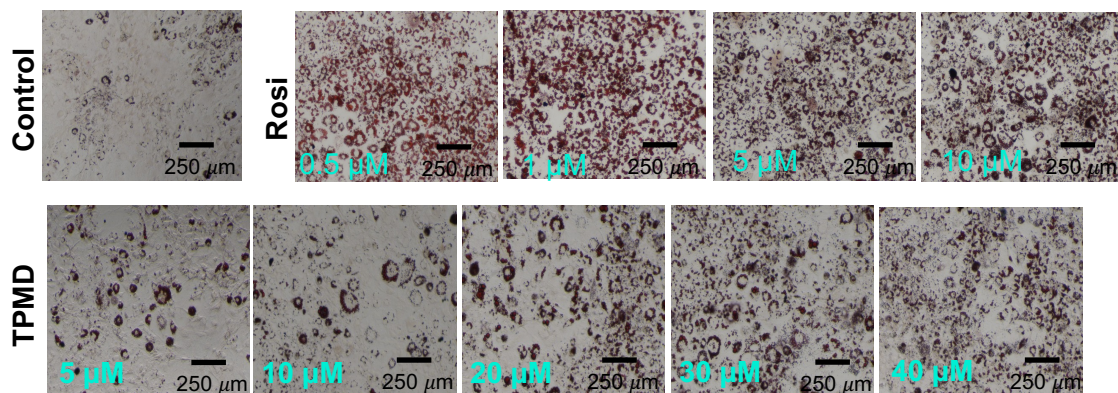

B

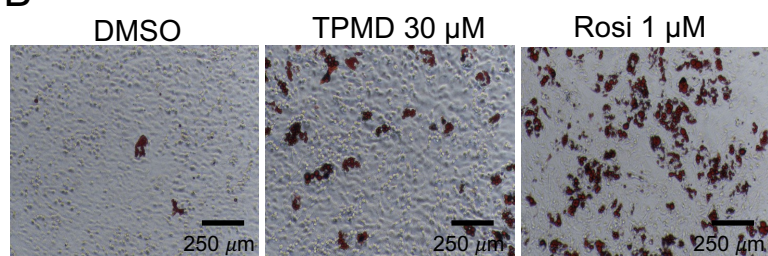

Preadipocytes isolated from SVF of eWAT

C

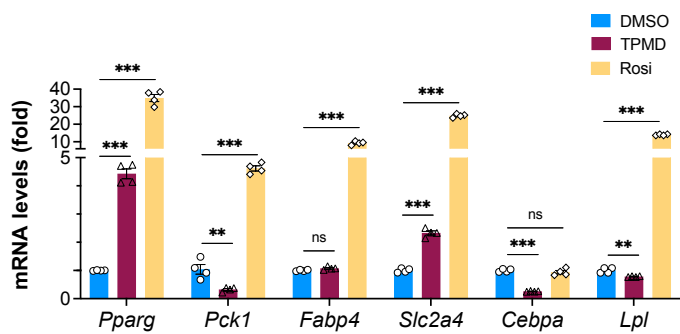

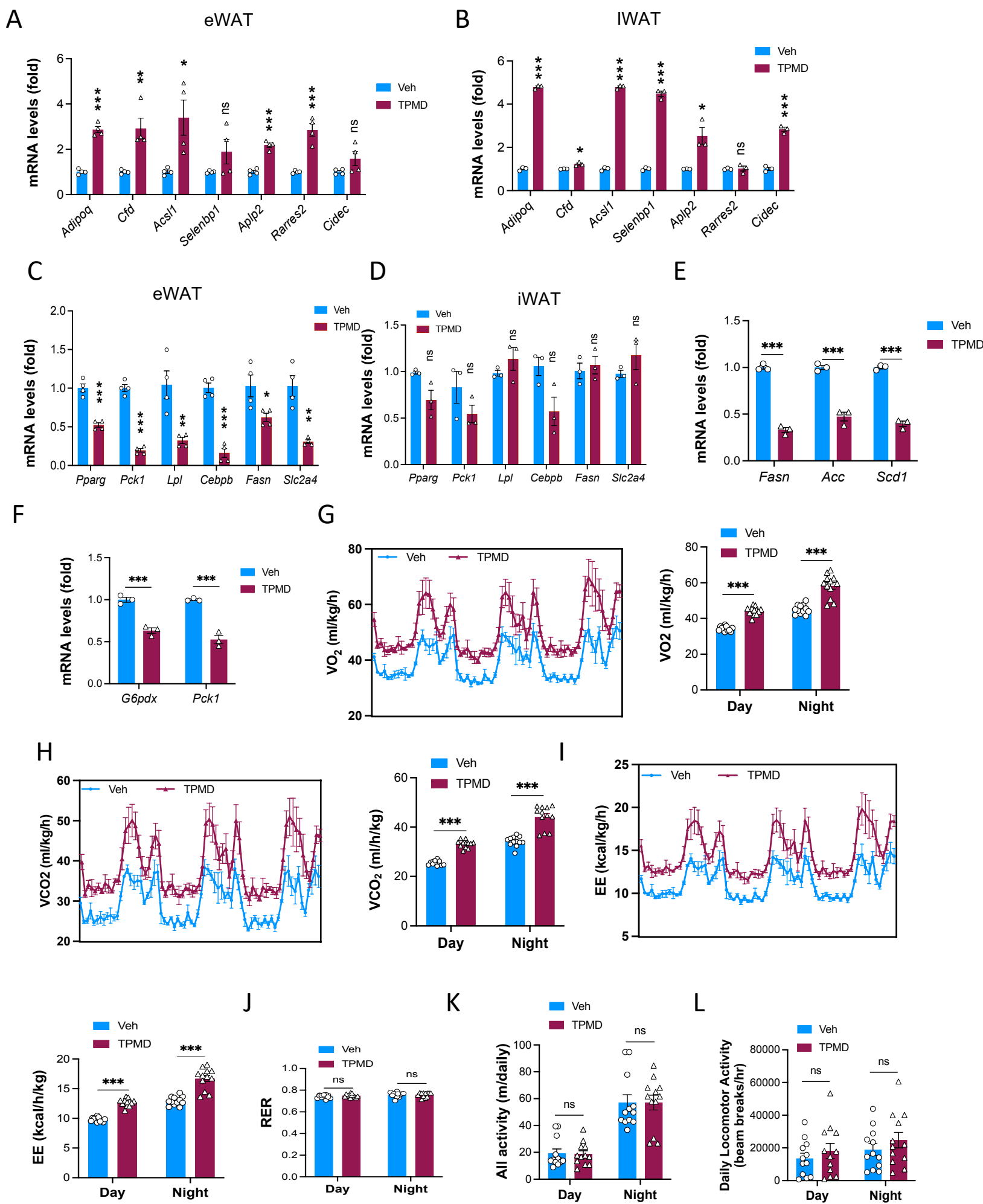

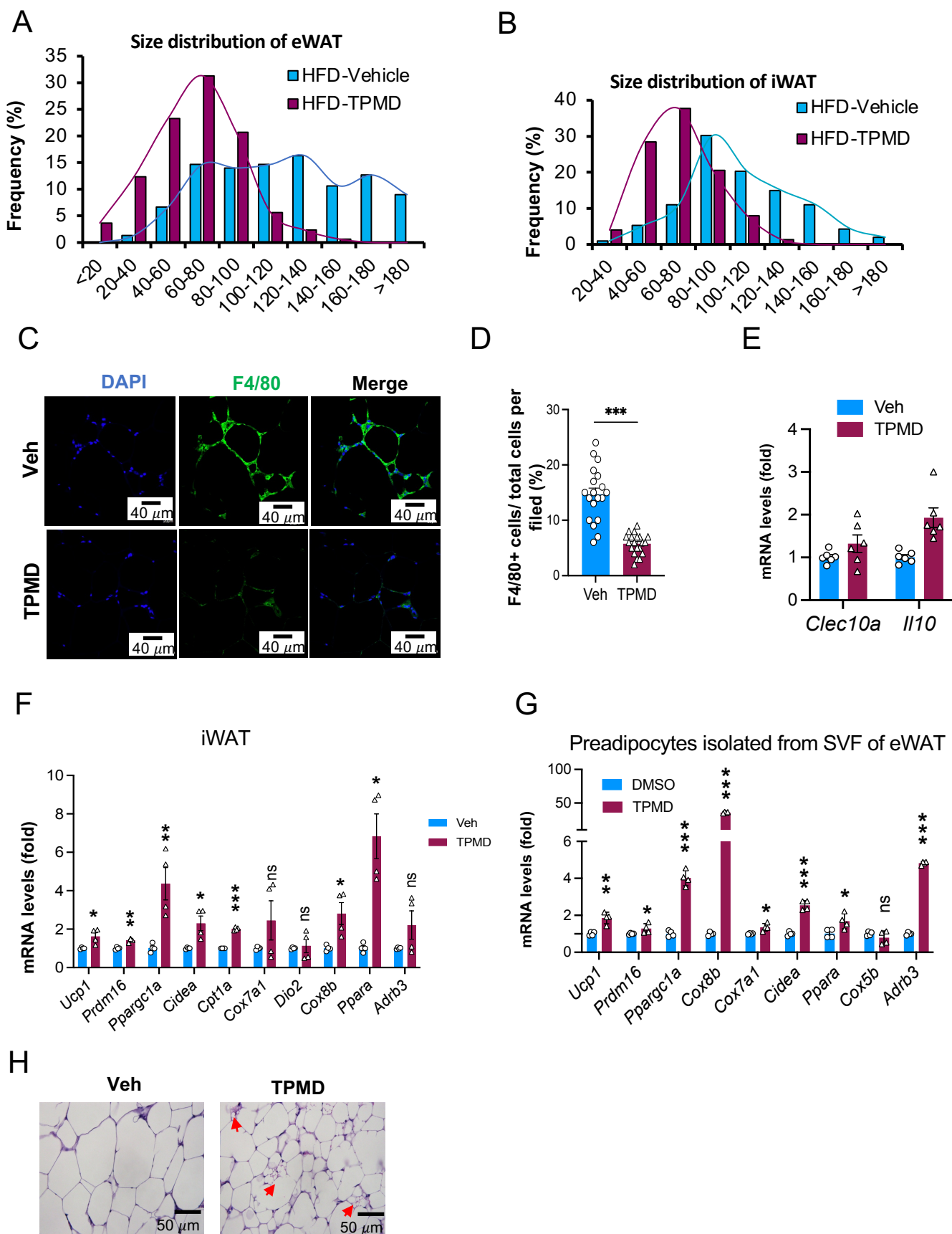

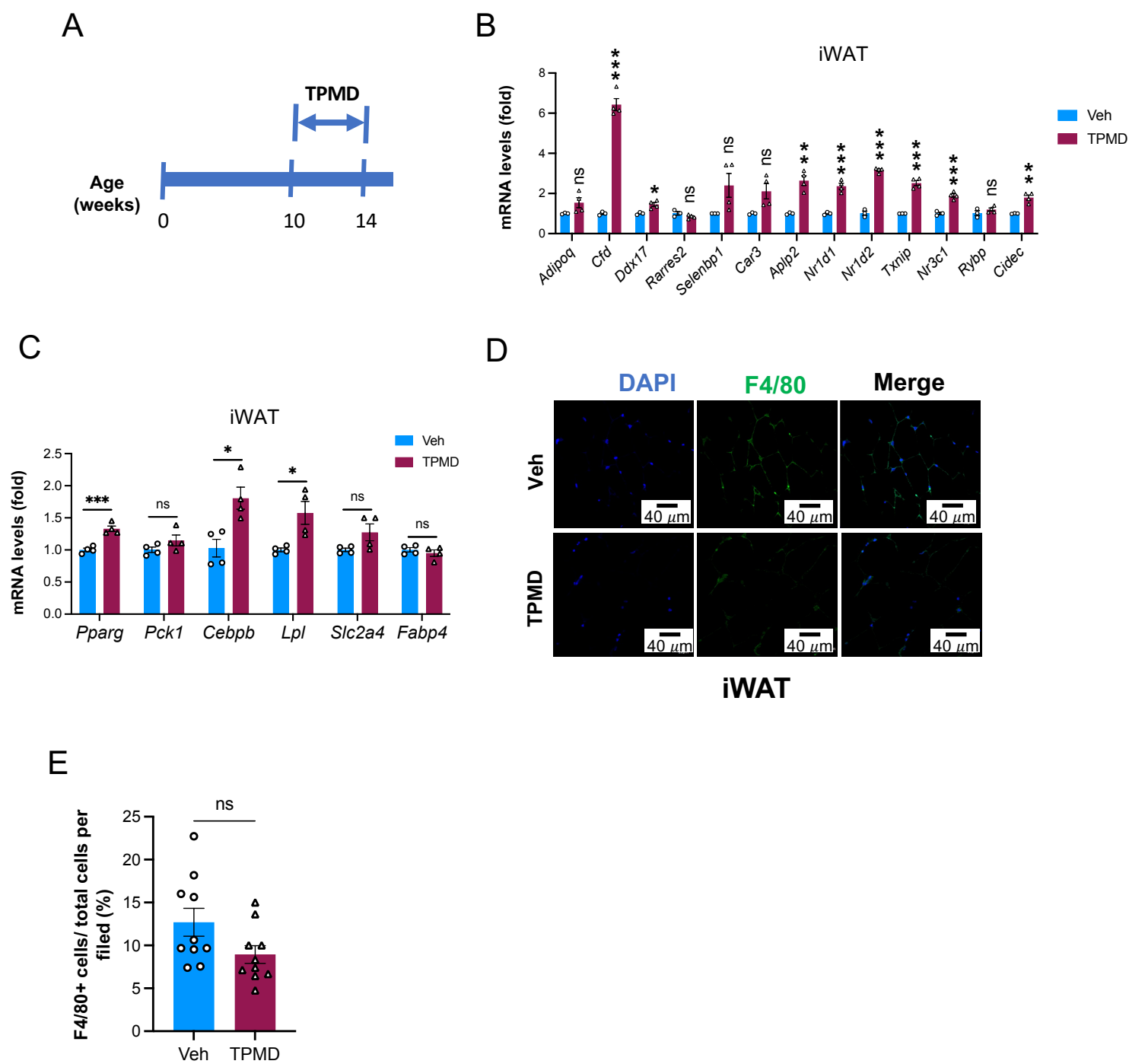

A

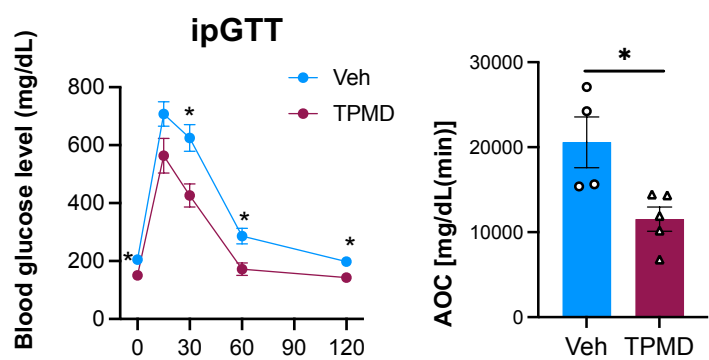

B

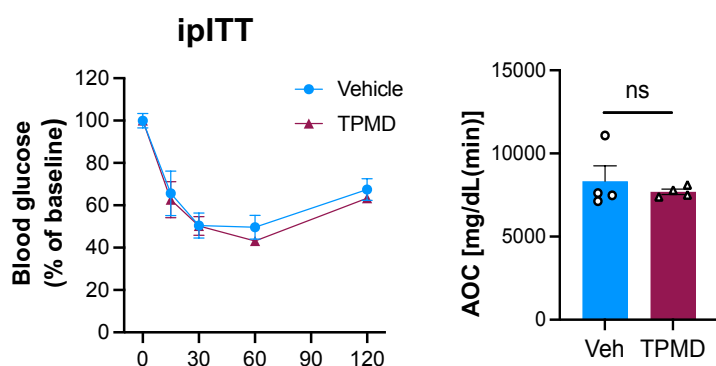

C

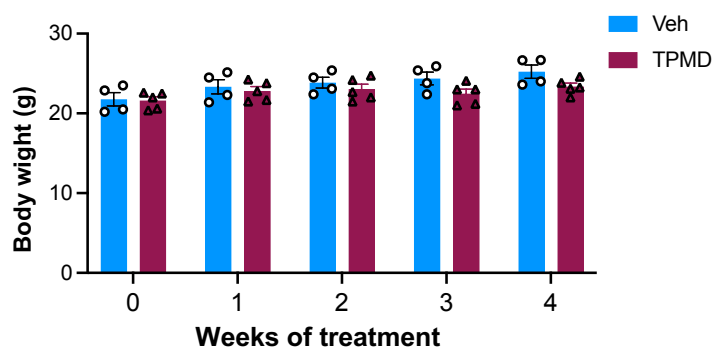

D

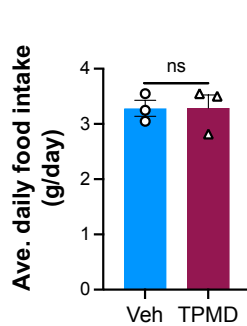

E

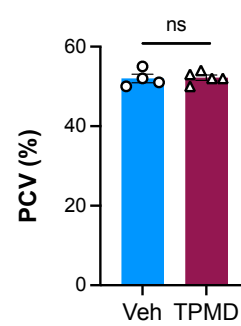

F

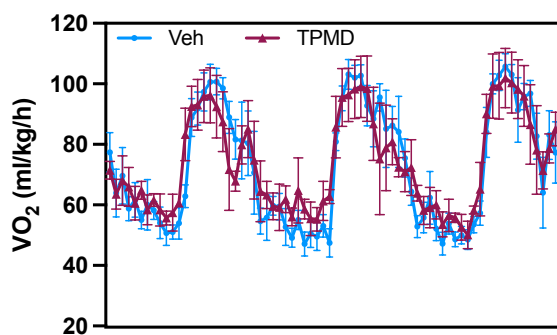

G

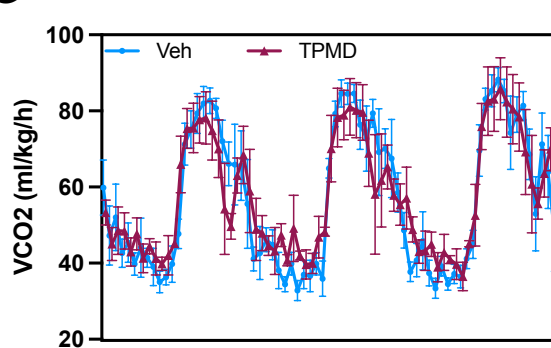

H

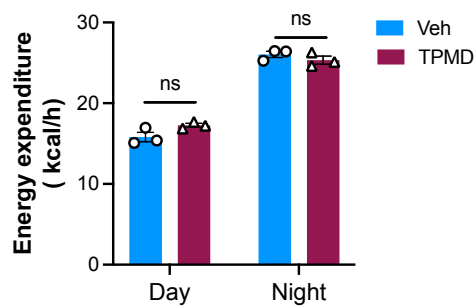

I

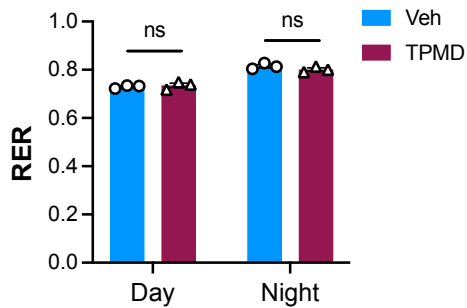

J

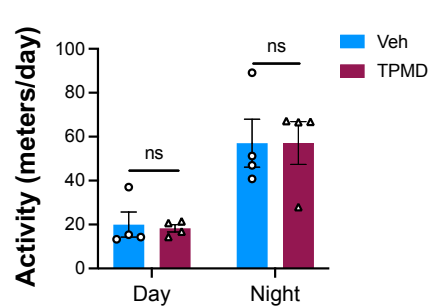

Fig. S7

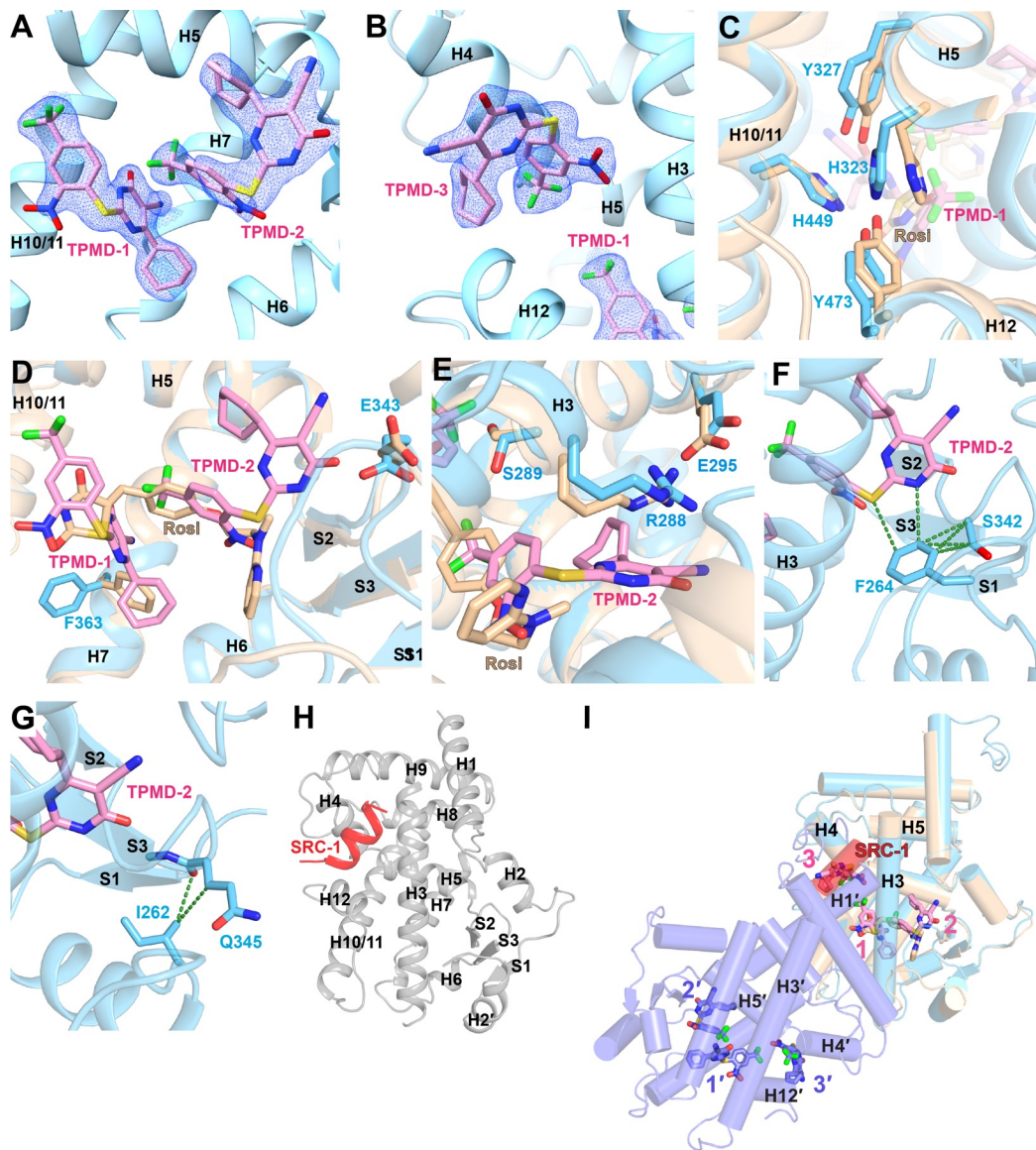

A

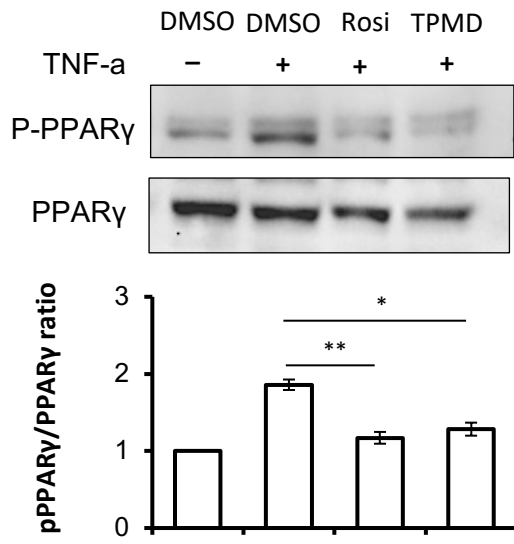

B

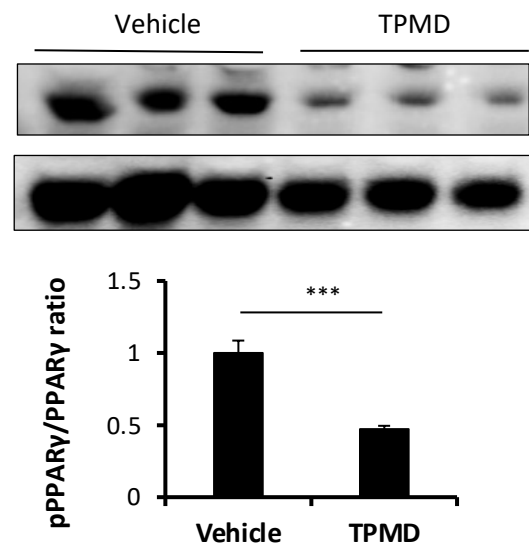

C

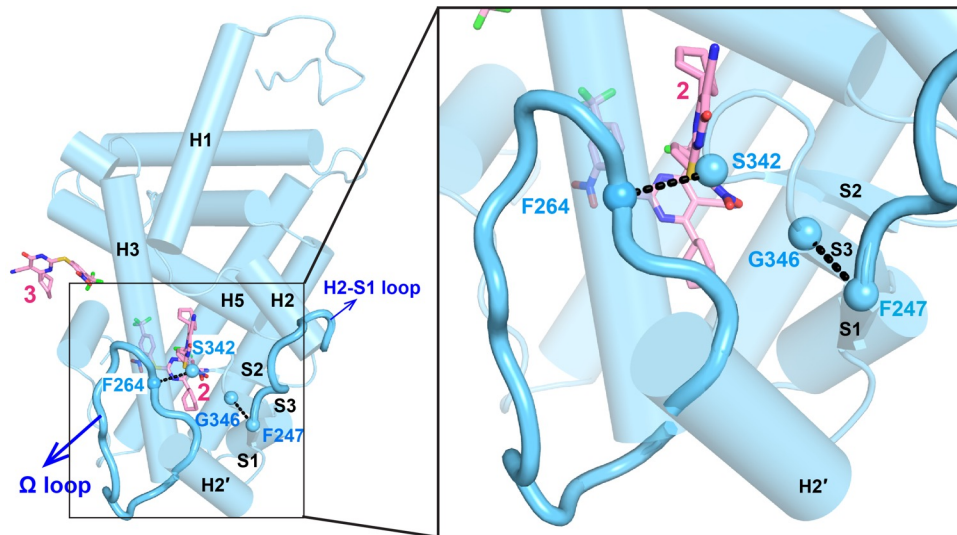

Supplement: Supplement 1 [file media-1.pdf]
