# Supplementary material for: Small molecule inhibitor of PPARγ acetylation promotes insulin sensitization and browning of white adipose tissue with improved safety
Source: bioRxiv. 2025 Dec 16:2025.12.15.694265. Preprint. [Version 1] doi: 10.64898/2025.12.15.694265 (PMC12746064; doi:10.64898/2025.12.15.694265)
Supplement: Supplement 2 [file NIHPP2025.12.15.694265v1-supplement-2.pdf]

bioRxiv preprint doi: https://doi.org/10.64898/2025.12.15.694265; this version posted December 16, 2025. The copyright holder for this preprint (which was not certified by peer review) is the author/funder, who has granted bioRxiv a license to display the preprint in perpetuity. It is made available under aCC-BY 4.0 International license.

Figure S1: TPMD protects pancreatic beta cells against ER stress.

A. Cell viability of INS-1 cells that were treated with TPMD at concentrations as indicated in the presence of Tm (0.1 μg/mL) for 72 h, determined by intracellular ATP assay. B. Live-cell phase-contrast images in INS-1 cells treated as in A. C. Immunoblotting of apoptotic markers in INS-1 cells that were treated with or without Tm (0.3 μg/ml) in the presence of TPMD at indicated concentrations or DMSO for indicated time points. Cleaved caspase-3 and PARP were determined by Western blotting. β actin was used as the loading control. D. Immunoblotting of ER stress markers in INS-1 cells that were treated with or without Tm (0.3 μg/ml) in the presence of TPMD at indicated concentrations or DMSO. β actin was used as a loading control. E-J. qRT-PCR analysis of relative mRNA expression levels of ER stress genes in INS-1 cells that were treated with or without Tm (0.3 μg/ml) in the presence of TPMD at indicated concentrations or DMSO. Data are presented as mean ± SEM. * $P < 0.05$, ** $P < 0.01$, and *** $P < 0.001$. ns for no significance.

bioRxiv preprint doi: https://doi.org/10.64898/2025.12.15.694265; this version posted December 16, 2025. The copyright holder for this preprint (which was not certified by peer review) is the author/funder, who has granted bioRxiv a license to display the preprint in perpetuity. It is made available under aCC-BY 4.0 International license.

Figure S2: TPMD shows weak activity in adipocyte differentiation.

A. 3T3-L1 adipocyte differentiation under the indicated treatment of TPMD or Rosi for last 4 days; Oil Red O staining for lipid accumulation at indicated concentrations. B. Primary mouse adipocyte differentiation with SVF isolated from eWAT under the indicated treatment of TPMD or Rosi for last 4 days. C. qRT-PCR analysis of relative mRNA expression levels of adipogenic genes in cells differentiated from SVF isolated from eWAT under the indicated treatment of TPMD or Rosi for last 4 days as in Fig. S2B. Data are presented as mean ± SEM.* $P < 0.05$, ** $P <0.01$, and *** $P <0.001$. ns for no significance.

Figure S3: TPMD improves insulin sensitivity and energy expenditure.

A-B. qRT-PCR analysis of mRNA levels of genes related with insulin sensitivity in eWAT (A) and in iWAT (B) from DIO mice treated with TPMD or vehicle. C-D. qRT-PCR analysis of mRNA levels of adipocyte housekeeping genes in eWAT (C) and in iWAT (D) from DIO mice treated with TPMD or vehicle. E-F. qRT-PCR analysis of mRNA levels of lipogenic (E) and gluconeogenic (F) markers in liver tissues from DIO mice treated with TPMD or vehicle. G-H. Oxygen (G) and carbon dioxide (H) consumption levels, normalized to body weight, in diet-induced obese (DIO) mice treated with TPMD or vehicle for 4 weeks. I. Energy expenditure levels, normalized by body weight. J. Respiratory exchange ratio (RER) measurement. K. Activity, expressed as distance traveled (m/mouse). Data are presented as mean ± SEM.* $P < 0.05$, ** $P <0.01$, and *** $P <0.001$.

Figure S4: TPMD promotes adipose tissue remodeling.

A-B. Adipocyte size distribution of eWAT (A) and iWAT (B) from DIO mice treated with TPMD or vehicle. C. Immunofluorescent staining of F4/80 in eWAT sections from DIO mice treated with

bioRxiv preprint doi: https://doi.org/10.64898/2025.12.15.694265; this version posted December 16, 2025. The copyright holder for this preprint (which was not certified by peer review) is the author/funder, who has granted bioRxiv a license to display the preprint in perpetuity. It is made available under aCC-BY 4.0 International license.

TPMD or vehicle for 4 weeks. D. Quantification of percentage of F4/80 positive cells in eWAT sections as in C. E. qRT-PCR analysis of mRNA levels of genes for M2 anti-inflammatory markers in eWAT from DIO mice treated with TPMD or vehicle. F. qRT-PCR analysis of mRNA levels of genes known for browning/thermogenesis in iWAT from DIO mice treated with TPMD or vehicle.

G. qRT-PCR analysis of mRNA levels of browning/thermogenic marker genes in adipocytes (differentiated from wild-type eWAT) under the indicated treatment of TPMD or DMSO. H. Representative images (arrows) of multilocular cells in eWAT sections from DIO mice treated with TPMD. Data are the mean± SEM. * P < 0.05, ** P <0.01, and *** P <0.001.

Figure S5: TPMD improves insulin sensitivity and comorbidity in Leptin-deficient obese mice.

A. Timing of i.p. drug administration. B-C. qRT-PCR analysis for mRNA levels of genes known for insulin sensitivity (B) and for adipocyte housing-keeping (C) in iWAT from *ob/ob* mice treated with TPMD or vehicle. D-E. Immunofluorescent staining of F4/80 in iWAT sections from *ob/ob* mice treated with TPMD or vehicle for 4 weeks; representative images (D) of F4/80 staining and quantification of percentage of F4/80 positive cells (E). F. H&E staining of liver sections from ob/ob mice treated with TPMD or vehicle for 4.5 weeks for both lower and higher magnifications. Data are the mean± SEM. * P < 0.05, ** P <0.01, and *** P <0.001.

Figure S6: TPMD has no obvious metabolic effects in male C57BL/6 mice fed a normal chow diet.

A. Glucose tolerance test from mice treated with TPMD or vehicle for 3 weeks. Blood glucose levels measured at indicated time points after intraperitoneal injection of glucose (2 g/kg body weight) following 14-h fasting and the AOC. B. Insulin tolerance test performed for mice treated with TPMD or vehicle for 3.5 weeks. Blood glucose levels normalized to basal level at indicated

bioRxiv preprint doi: https://doi.org/10.64898/2025.12.15.694265; this version posted December 16, 2025. The copyright holder for this preprint (which was not certified by peer review) is the author/funder, who has granted bioRxiv a license to display the preprint in perpetuity. It is made available under aCC-BY 4.0 International license.

time points after intraperitoneal injection of insulin (0.7 IU/kg body weight) following 6-h fasting and the AOC. C. Body weight of mice treated with TPMD or vehicle for 4 weeks. D. Daily food intake, measured for three consecutive days during the 4th week of treatment. Changes in body weight of ob/ob mice treated with TPMD or vehicle over the period of 4-wk treatment. E. Measurement of PCV in mice treated with TPMD and vehicle. (F-I). Metabolic cage analysis of ND mice treated with TPMD or vehicle after 4-week treatment, Measurement of oxygen (F) and carbon dioxide (G) consumption levels. H. Energy expenditure levels. (I) Respiratory exchange ratio (RER) measurement. (J). Locomotor activity, expressed as distance traveled (m/mouse). Data are the mean± SEM. * $P < 0.05$, ** $P < 0.01$, and *** $P < 0.001$.

Figure S7: Additional structural features of the PPARγ LBD in complex with TPMD.

The $2F_O - F_C$ electron density map (1.0 σ) of TPMD-1, TPMD-2 (A) and TPMD-3 (B) were displayed at a radius of 2.0 Å to the TPMDs. (C-E) Conformational changes of PPARγ LBD induced by TPMD-1 (C and D) and TPMD-2 (D and E), in comparison with Rosi-bound PPARγ LBD. The overall structure of Rosi-bound LBD is shown in beige ribbons while its protein residues and Rosi are shown in stick representation with carbon atoms depicted in beige. Helix 3 was hidden for better display of TPMDs in Figure B and D. (F-G) Interactions of the Ω loop with TPMD-2 and the β turn between β2 and β3 strands. (H) An overall structure of the PPARγ LBD containing SRC-1. The LBD and SRC-1 are shown in grey and red ribbons respectively. (I) TPMD-3 functions as a lattice contact between two crystallographic neighboring LBD molecules, colored with light blue and light purple respectively. The LBD in light blue is in the same orientation as in Fig. 6A. TPMD-1, 2 and 3 shown in pink sticks belong to the LBD in light blue while TPMD-1^S, 2^S and 3^S shown in purple sticks belong to the crystallographically symmetric neighboring LBD in light purple. Rosi-bound PPARγ LBD (beige) in complex with SRC-1 (red) is superimposed with TPMD-bound LBD (blue).

bioRxiv preprint doi: https://doi.org/10.64898/2025.12.15.694265; this version posted December 16, 2025. The copyright holder for this preprint (which was not certified by peer review) is the author/funder, who has granted bioRxiv a license to display the preprint in perpetuity. It is made available under aCC-BY 4.0 International license.

Figure S8: Effect of TPMD on PPARγ PTMs

A-A'. Phosphorylation of PPARγ S273 in 3T3-L1 adipocytes under the treatment of TPMD 20 μM or Rosi 10 μM for 6 h and for TNFα 10 ng/ml for 1 h before harvest; pSer273 of PPARγ was detected by Western blotting using PPARγ pSer273-specific antibody (A) and quantified by image-J (A'). B-B'. Immunoblotting (B) and quantification (B') for phosphorylation status of PPARγ protein at S273 in eWAT from DIO mice treated with TPMD or vehicle for 4. weeks. Data are the mean± SEM and are representative of 3 independent experiments. * $p<0.05$, ** $p<0.01$, *** $p<0.001$, n=4. (**C**) A cartoon representation of PPARγ LBD in complex of TPMD molecules to highlight the Cα of mutated residues in double mutants F275C/G374C (F247C/G346C in PPARγ1) and F292C/S370C (F264C/S342C in PPARγ1). The Cα of F275, G374, F292, S370 are shown in spheres. The Ω loop and H2-S1 loop are highlighted using thicker loops.

bioRxiv preprint doi: https://doi.org/10.64898/2025.12.15.694265; this version posted December 16, 2025. The copyright holder for this preprint (which was not certified by peer review) is the author/funder, who has granted bioRxiv a license to display the preprint in perpetuity. It is made available under aCC-BY 4.0 International license.

Table S1

Primers used in this work

Gene	Forward Primer	Reverse Primer

bioRxiv preprint doi: https://doi.org/10.64898/2025.12.15.694265; this version posted December 16, 2025. The copyright holder for this preprint (which was not certified by peer review) is the author/funder, who has granted bioRxiv a license to display the preprint in perpetuity. It is made available under aCC-BY 4.0 International license.

Tbp	ACCCTTCACCAATGACTCCTATG	TGACTGCAGCAAATCGCTTGG
Gapdh	AAAGACTGGAGCCCCACACTCTAC	ATCCCGTATTTCACCTCTGCTTC
Pparg	GCATGGTGCCTTCGCTGA	TGGCATCTCTGTGTCAACCATG
Fabp4	AAGGTGAAGAGCATCATAACCCT	TCACGCCTTTCATAACACATTCC
Cebpa	CAAGAACAGCAACGAGTA	GTCACTGGTCAACTCCAGCAC
Slc2a4	GTGACTGGAACACTGGTCCTA	CCAGCCACGTTGCATTGTAG
Fasn	GCTGGCATTCGTGATGGAGTCGT	AGGCCACCAGTGATGATGTAACTCT
Lpl	GGGAGTTTGGCTCCAGAGTTT	TGTGTCTTCAGGGGTCCTTAG
Adipoq	TGTTCCTCTTAATCCTGCCCA	CCAACCTGCACAAGTTCCCTT
Cfd	CATGCTCGGCCCTACATGG	CACAGAGTCGTCATCCGTCAC
Car3	TGACAGGTCTATGCTGAGGGG	CAGCGTATTTTACTCCGTCCAC
Rarres2	GCCTGGCCTGCATTAAAATGG	CTTGCTTCAGAATTGGGCAGT
Selenbp1	ATGGCTACAAAATGCACAAAGTG	CCTGTGTTCCGGTAAATGCAG
Ddx17	TCTTCAGCCAACAATCCCAATC	GGCTCTATCGGTTTCACTACG
Cyp2f2	GTCGGTGTTCACGGTGTACC	AAAGTTCCGCAGGATTTGGAC
Ucp1	CACCTTCCCCCTGGACACT	CCCTAGGACACCTTTATACCT
Prdm16	AGGAGGAGGAGAGAGATTCCG	GTCCGGGTCAGGTTCATACAT
Ppargc1a	CCCTGCCATTGTTAAGACC	TGCTGCTGTTCCTGTTTTC
Cidea	ATCACAACTGGCCTGGTTACG	TACTACCCGGTGTCCATTTCT
Cox5b	TGCTACCTCCAAAGGCAGCTTC	CATCGCTGACTCTCGCCTTTGT
Cox7a1	CAGCGTCATGGTCAGTCTGT	AGAAAACCGTGTGGCAGAGA
Cox8b	GAACCATGAAGCCAACGACT	GCGAAGTTCACAGTGGTTCC
Ppara	TTCCCTGTGAACTGACGTTT	CCACCATGTTGGATGGATGTG
Dio2	AGAGTGGAGGCGCATGCT	GGCATCTAGGAGGAAGCTGTT
Adrb3	CCTTCCGTCGTCTTCTGTGT	AGCCATCAAACCTGTTGAGC

bioRxiv preprint doi: https://doi.org/10.64898/2025.12.15.694265; this version posted December 16, 2025. The copyright holder for this preprint (which was not certified by peer review) is the author/funder, who has granted bioRxiv a license to display the preprint in perpetuity. It is made available under aCC-BY 4.0 International license.

Tmem26	TTCCTGTTGCATTCCCTGGTC	GCCGGAGAAAGCCATTTGT
Tnf α	CTGAACTTCGGGGTGATCGG	CTACGACGTGGGCTACAGG
Il1b	TGCCACCTTTTGACAGTGATG	TGATGTGCTGCTGCGAGATT
Il6	CACTTCACAAGTCGGAGGCT	CTGCAAGTGCATCATCGTTGT
Ccl2	AGGTCCCTGTCATGCTTCTG	TCTCCAGCCTACTCATTGGGA
Clec10a	CAGATCTGGGGCCGTCAAG	GGGAGGAAATGCATCTGGGT
Il10	AGGCGCTGTCATCGATTTCT	ATGGCCTTGTAGACACCTTGG
Adgre1	TCTGCTTCTGTACAGCCACG	CCTCAGAACCCACAGTGTCC
Cd68	TGTTCAGCTCCAAGCCCAAA	GTACCGTCACAACCTCCCTG
Pck1	TGAAAGGCCGCACCATGTAT	GGGCGAGTCTGTCAGTTCAA
H6pd	ATCCGGGGCATCTACAATG	TGGCAAAGGGTGTAGTGTCA
Acc	GCCTCTTCCTGACAAACGAG	TGACTGCCGAAACATCTCTG
Scd1	GAGGCCTGTACGGGATCA	GCCCAGTCGTACACGTCA
Myh7	TTGCTACCCTCAGGTAGGAGT	GGTACTACATGCCAGAAGCCC
Nppb	TCTCAAGCTGCTTTGGGCAC	AGCCAGGAGGTCTTCCTACAA
Col1a1	CTGACGCATGGCCAAGAAGA	ATACCTCGGGTTTCCACGTC
Bglap2	GTCCAAGCAGGAGGGCAATA	AGGCGGTCTTCAAGCCATAC
Spp1	CTTGCTTGGGTTTGCAGTCTT	TCACTGCCAATCTCATGGTCG
Alpl	GTGGTGGAAGACCGTGACTAC	TCGGGGGAACTTTAACATCGT

Table S2. Diffraction Data Statistics

Dataset	PPARγ LBD in complex with TPMD	PPARγ LBD in complex with SRC-1
Beamline	NYX 19-ID	NYX 19-ID
Wavelength (Å)	0.9795	0.9795
Space group	$P2_12_12_1$	$P2_12_12$
Unit cell parameters A, b, c (Å)	70.07, 70.35, 72.17	130.93, 53.86, 54.06
α, β, γ (°)	90, 90, 90	90, 90, 90

bioRxiv preprint doi: https://doi.org/10.64898/2025.12.15.694265; this version posted December 16, 2025. The copyright holder for this preprint (which was not certified by peer review) is the author/funder, who has granted bioRxiv a license to display the preprint in perpetuity. It is made available under aCC-BY 4.0 International license.

Z_a [a]	1	1
Bragg spacings (Å) [b]	40.90-1.99 (2.11-1.99)	49.97-2.20 (2.32-2.20)
Total reflections	594443 (15894)	216215 (11199)
Unique reflections	16959 (848)	16517 (826)
Completeness (%)	94.1 (81.8)	93.6 (60.4)
Multiplicity	35.1 (18.7)	13.1 (13.6)
$CC_{1/2}$ (%) [c]	99.2 (31.0)	99.8 (50.7)
$\langle I/\sigma(I) \rangle$ [d]	11.2 (1.6)	13.9 (1.5)
R_{merge} [e]	0.317 (2.364)	0.102 (1.919)
R_{pim} [f]	0.053 (0.556)	0.029 (0.536)
R_{work} [g]	0.2039	0.2200
R_{free} [h]	0.2398	0.2605
RMS bond deviation (Å)	0.002	0.003
RMS angle deviation (º)	0.600	0.582
Average B factor (Å^2)	50.03	67.03
Ramachandran analysis favored/allowed (%)	96.84/99.65	97.74/100.00
PDB code	9ZL6	9ZL5

[a] Z_a stands for number of subunits per asymmetric unit.
[b] Values in the outermost shell are given in parentheses.
[c] $CC_{1/2}$ is the correlation coefficient of integrated intensities between randomly split two half data sets.
[d] $\langle I/\sigma(I) \rangle = \langle (\langle Ii \rangle) / \langle \sigma(\langle Ii \rangle) \rangle$
[e] $R_{merge} = (\Sigma |Ii - \langle Ii \rangle|) / \Sigma |Ii|$, where Ii is the integrated intensity of a given reflection.
[f] $R_{pim} = (1 / (n - 1))^{1/2} \times (\Sigma |Ii - \langle Ii \rangle|) / \Sigma |Ii|$, where Ii is the integrated intensity of a given reflection.
[g] $R_{work} = (\Sigma | |Fo| - |Fc| |) / \Sigma |Fo|$, where Fo and Fc denote observed and calculated structure factors, respectively.
[h] R_{free} was calculated using 10% of data excluded from refinement.
